# *StuPPO9* Enhances Drought Tolerance in Potato (*Solanum tuberosum*) by Coordinating Photosynthetic Stability and Secondary Metabolism Reprogramming

**DOI:** 10.3390/plants15162495

**Published:** 2026-08-18

**Authors:** Ming Chi, Bo Liu, Hengzhao Yang, Jingting Chen, Yue Liu

**Affiliations:** College of Horticulture and Landscape, Tianjin Agricultural University, No. 22 Jinjing Road, Tianjin 300392, China

**Keywords:** potato, drought stress, *StuPPO9*, polyphenol oxidase, physiological response, transcriptomics

## Abstract

Drought stress is a primary abiotic constraint limiting potato productivity. While polyphenol oxidase (PPO) is known to participate in stress responses, the specific role of *StuPPO9* in drought tolerance remains poorly understood. In this study, we generated *StuPPO9*-overexpressing (OE) and CRISPR/Cas9-mediated knockout (C4) lines in the potato cultivar ‘Atlantic’. Under sustained drought stress, OE lines exhibited significantly superior growth phenotypes compared to wild-type (WT) and C4 plants, characterized by increased leaf and root relative water content, root number and enhanced photosynthetic efficiency (*Pn* and *Gs*). OE plants also maintained lower levels of MDA and ROS through elevated antioxidant enzyme activities. Notably, transcriptomic analysis revealed that *StuPPO9* triggers a global reprogramming of metabolic pathways. Key drought-responsive genes associated with phenylpropanoid biosynthesis (e.g., anthocyanin acyltransferase), terpenoid metabolism, and hormone signaling (e.g., HPt protein) were significantly upregulated in OE plants. These findings suggest that *StuPPO9* confers drought resilience through a multi-layered network involving optimized carbon allocation, reinforced cell wall integrity, and enhanced ROS scavenging capacity. This study provides a promising genetic target and theoretical foundation for breeding drought-resistant potato varieties.

## 1. Introduction

Drought is one of the major abiotic stresses that limit crop growth and yield. Potato (*Solanum tuberosum* L.) is an important global food crop with a relatively shallow root system, making it particularly sensitive to water stress. Drought impedes potato growth and development, reduces photosynthetic efficiency, exacerbates oxidative damage, and ultimately leads to significant reductions in tuber yield and quality [1,2]. Therefore, identifying key drought-resistance genes in potato and elucidating their functional mechanisms is crucial for breeding drought-resistant varieties and ensuring food security [3,4].

Through long-term evolution, plants have developed complex stress response mechanisms, including morphological adjustments [5], physiological and biochemical adaptations [6], and gene expression reprogramming [7]. Polyphenol oxidase (PPO) is a copper-containing metalloenzyme widely distributed in plants. Traditional studies primarily focused on its role in catalyzing the oxidation of phenolic compounds, resulting in browning of fruits and vegetables [8]. However, increasing evidence suggests that *PPO* can be induced by various biotic stresses and hormone signals, actively participating in plant stress responses [9]. For example, under salt stress (100 mM NaCl), drought stress (7 days of water deprivation), or inoculation with *Pseudomonas koreensis*, PPO activity significantly increases [10]. In rice, transgenic *Arabidopsis thaliana* plants expressing a fusion of the *OsPPO* promoter with the *GUS* gene (*OsPPO::GUS*) exhibited high expression under drought conditions [11]. PPO increases phenolic compound production in plants, enhancing their reactive oxygen species (ROS) scavenging capacity and improving tolerance to abiotic stresses. Akhtar et al. characterized the response of the *PPO* promoter to drought by measuring relative GUS activity in wild-type and *OsPPO::GUS* transgenic *Arabidopsis* [12]. In tobacco, overexpression of the *Morus* *MnMYB3R* gene upregulated *NtPPO2* expression, thereby enhancing PPO activity and improving drought tolerance [13].

The potato genome contains nine *PPO* genes (*StuPPO1–9*) [14]. Among these, *StuPPO9* forms a distinct evolutionary branch, and its promoter region is enriched with stress- and hormone-responsive *cis*-elements, suggesting a specific role in abiotic stress responses [9]. Preliminary studies confirmed that *StuPPO9* overexpression enhances resistance of *Nicotiana benthamiana* to late blight [9]. However, the function, specific roles, and molecular mechanisms of *StuPPO9* in potato drought tolerance remain unclear.

In this study, the potato cultivar ‘Atlantic’ was used to generate stable transgenic lines overexpressing *StuPPO9* (OE) and knockout lines (C4) using CRISPR/Cas9 technology through Agrobacterium-mediated genetic transformation. By simulating drought stress conditions, differences among transgenic and wild-type (WT) plants were systematically compared regarding growth, photosynthetic physiology, antioxidant systems, and osmotic regulation. Combined with transcriptome sequencing (RNA-seq), gene expression changes regulated by *StuPPO9* under drought stress were analyzed, and key differentially expressed genes (DEGs) were identified. This study aims to elucidate the physiological and molecular mechanisms by which *StuPPO9* regulates drought tolerance in potato, providing theoretical support for utilizing this gene in breeding drought-resistant potato varieties.

## 2. Results

### 2.1. Molecular Identification of Transgenic Potato Plants

**Figure 1:**
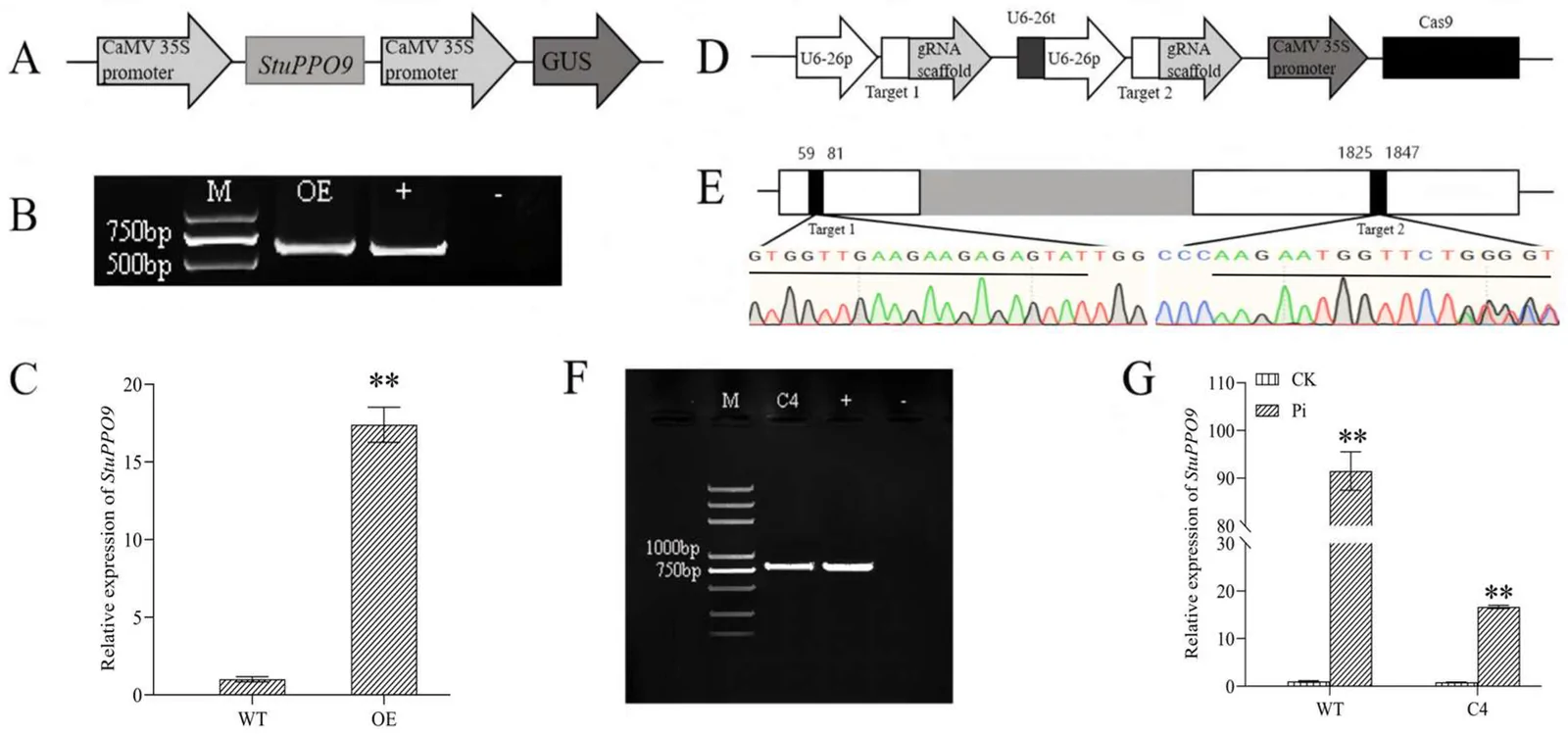
Positive identification of transgenic potato plants. (**A**) PCAMBIA1301S-*StuPPO9* overexpression vector; (**B**) PCR products of *GUS* gene in plants (M: Trans2k Plus DNA Marker; OE: *GUS* gene amplification product in OE plants; +: positive plasmid control; −: WT); (**C**) *StuPPO9*-related expression levels in OE plants (** indicates highly significant difference at *p* < 0.01); (**D**) Schematic of CRISPR/Cas9 construct; (**E**) Representative sequences of CRISPR mutants induced in WT and target genes (sgRNA sequences underlined); (**F**) PCR identification of *Hyg* gene in CRISPR plants (M: Trans2k Plus DNA Marker; C4: *Hyg* gene amplification product from CRISPR plants; +: positive plasmid control; −: WT); (**G**) *StuPPO9*-related expression levels in CRISPR plants (CK: healthy control without infection; Pi: 3 days after infection; ** indicates highly significant difference at *p* < 0.01).

To investigate the function of *StuPPO9*, transgenic potato plants were generated via *Agrobacterium*-mediated transformation. PCR and sequencing analysis confirmed the successful integration of the pCAMBIA1301S-*StuPPO9* vector in overexpression (OE) lines and the presence of targeted mutations in CRISPR/Cas9-mediated knockout lines (Figure 1A,D).

In the OE lines, positive transformants were identified by the amplification of the *GUS* reporter gene (Figure 1B). RT-qPCR analysis revealed that under basal conditions, the expression level of *StuPPO9* in OE plant was significantly higher than the one in wild-type (WT) plant (Figure 1C), confirming successful constitutive overexpression.

For the CRISPR/Cas9-edited lines (C4), positive transformants were verified by PCR amplification of the *Hyg* marker gene, yielding the expected target bands between 750 bp and 1000 bp (Figure 1F). Sanger sequencing and chromatogram analysis at the dual-target sites (Target 1 and Target 2) of the *StuPPO9* locus confirmed successful gene editing events (Figure 1E), which disrupted the open reading frame. To further evaluate the editing patterns and cleavage efficiency at the target locus, 15 individual monoclonal colonies derived from line C4 were subjected to Sanger sequencing and alignment analysis. A total of five distinct editing types were identified, including single-nucleotide, di-nucleotide, and multi-nucleotide deletions, as well as nucleotide substitutions (Figure S1). Notably, three sequences remained unedited and retained the wild-type sequence, indicating an overall gene editing efficiency of 80% (12/15) in this line, alongside the presence of somatic chimerism commonly observed during tissue culture regeneration in tetraploid potato. Furthermore, RT-qPCR analysis demonstrated that under *Phytophthora infestans* induction (Pi) conditions, the transcription level of *StuPPO9* was drastically upregulated in WT plants but remained markedly suppressed in C4 plants, reaching only 18.21% of the induced WT levels (Figure 1G). Combined with the significant overexpression of *StuPPO9* confirmed in the OE lines (Figure 1B,C), these molecular data collectively validate the stable generation of *StuPPO9*-OE and C4 potato lines for subsequent physiological and stress response studies.

### 2.2. Phenotypic Changes in Transgenic Potato Plants Under Drought Stress

**Figure 2:**
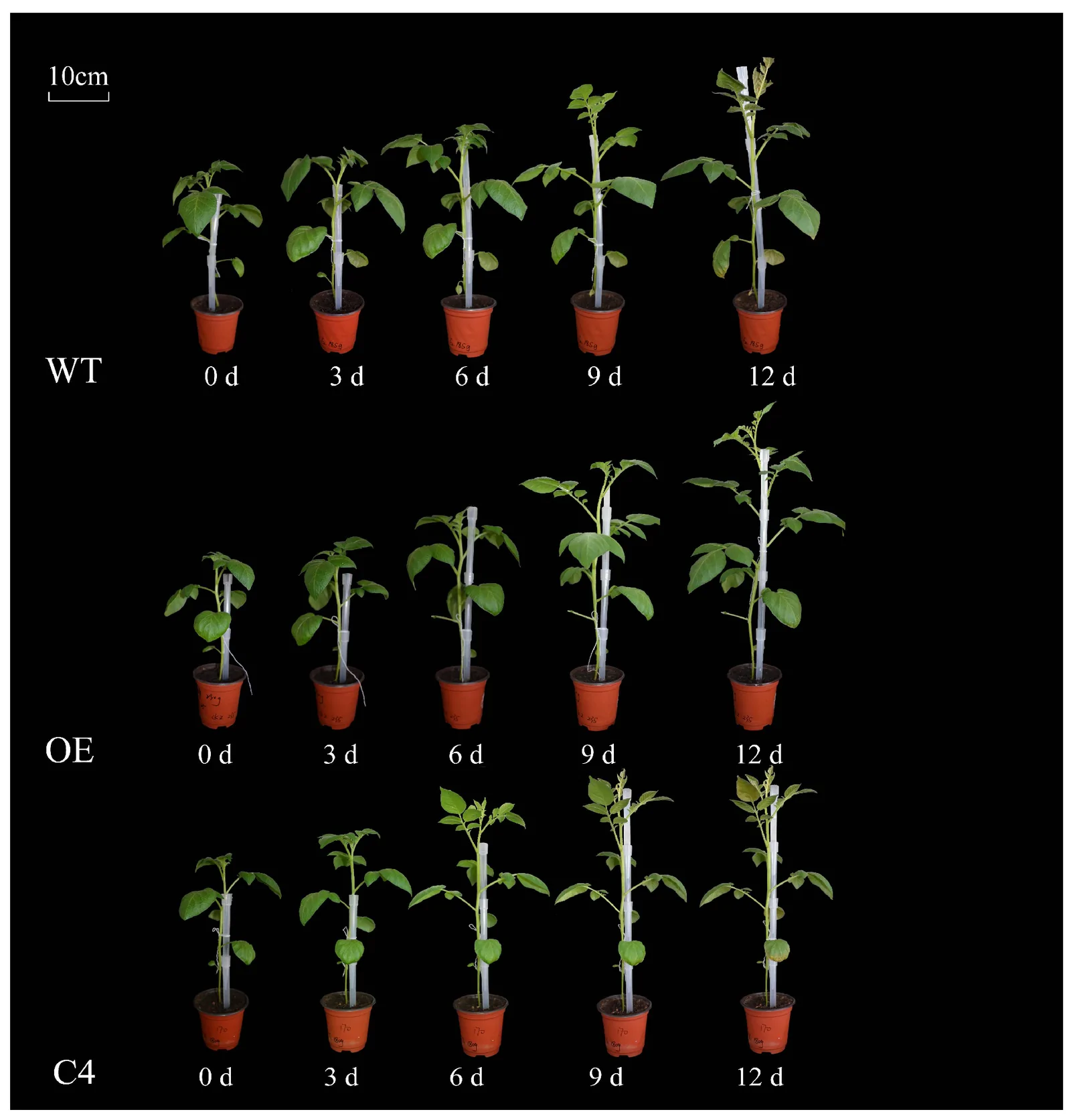
Phenotypic changes in shoots of *StuPPO9* transgenic potatoes under drought stress.

After 12 days of progressive drought stress, all genotypes exhibited varying degrees of growth inhibition (Figure 2). WT and C4 plants displayed severe phenotypic symptoms, including pronounced wilting, chlorosis of the lower leaves, and nearly complete cessation of apical growth. In contrast, OE lines maintained superior vigor, with most leaves remaining green and only slight wilting observed in the basal foliage (Figure 2).

**Figure 3:**
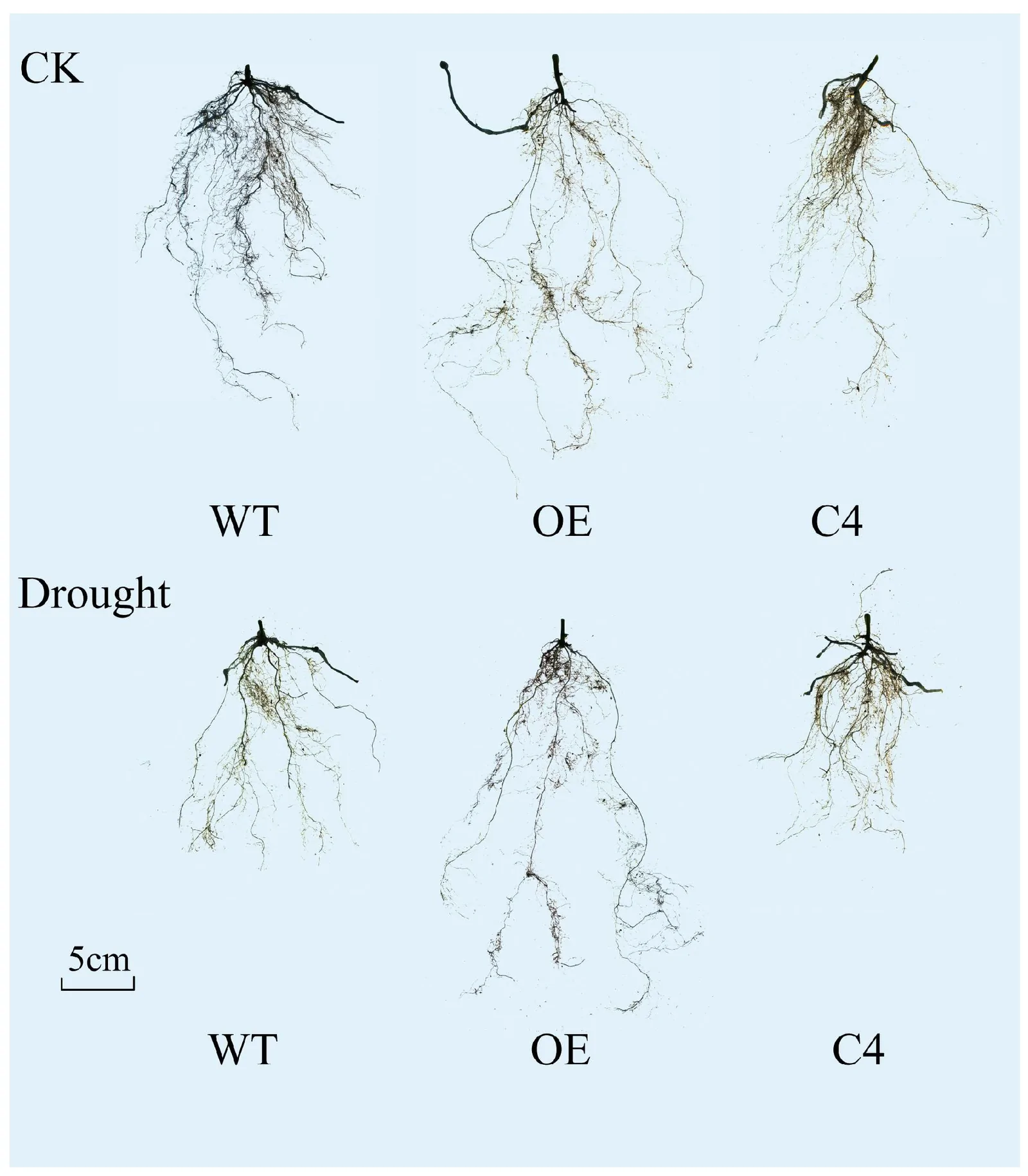
Phenotypic changes in roots of *StuPPO9* transgenic potatoes under drought stress.

To evaluate the impact of *StuPPO9* on root development, we quantified primary and lateral root architecture under both control (CK) and drought conditions (Table 1 and Figure 3). Under well-watered (CK) conditions, all genotypes developed a comparable primary root framework, whereas distinct genotypic variations were observed in lateral branching: WT developed the most dense lateral root network (21.33 ± 1.45), followed by C4 (16.67 ± 1.20), while the *StuPPO9*-overexpressing (OE) lines exhibited the fewest lateral roots (14.67 ± 0.88). Although drought stress universally restricted root development across all genotypes, this repressive effect was significantly mitigated in OE plants. Under drought conditions, the OE lines demonstrated greater resilience by maintaining a more robust root system, exhibiting a significantly higher number of primary roots (3.33 ± 0.33) compared to WT (2.67 ± 0.33) and C4 (2.33 ± 0.33), while preserving lateral root numbers (10.67 ± 1.20) at a level comparable to WT (10.33 ± 0.88). Conversely, the C4 knockout lines were highly sensitive to water deficit, with their lateral root number dramatically reduced to only 8.67 ± 0.67, and their primary root development nearly entirely inhibited by day 12 of drought. These results collectively demonstrate that overexpressing *StuPPO9* enhances drought tolerance in potato by preserving primary root growth and maintaining overall root system architecture under water deficit conditions.

Under drought stress, WT plants exhibited an intermediate growth phenotype; specifically, their increments in plant height, stem diameter, and leaf area were significantly lower than those of OE plants, yet remained significantly higher than those of the C4 line. The inhibitory effect of drought was most pronounced in the leaf area, which was reduced by 27.41%, 53.18%, and 60.73% in OE, WT, and C4 plants, respectively, compared to their corresponding controls. Similarly, OE plants maintained superior growth stability in terms of plant length and stem diameter, showing the smallest reductions (30.29% and 16.98%) among the three genotypes, whereas C4 plants suffered the most severe growth retardation (49.34% and 41.61%). Collectively, these results demonstrate that *StuPPO9* overexpression significantly confers enhanced drought tolerance by mitigating the suppression of vegetative growth (Table 1).

Drought stress significantly reduced the leaf and root relative water content (RWC) across all tested potato genotypes, though the rate of decrease was markedly higher in WT than in OE lines, and highest in the C4 knockout line (Table 1). Specifically, after drought exposure, OE plants maintained a leaf RWC of 91.55%, whereas WT and C4 leaves fell significantly lower to 90.45% and 87.44%, respectively. A similar trend was observed in the roots, with OE, WT, and C4 maintaining RWCs of 92.76%, 91.24%, and 89.10%, correspondingly. In comparison to WT plants under drought, the OE lines exhibited significantly elevated water levels in both leaves and roots, while C4 plants showed the opposite. Overall, these findings indicate that *StuPPO9* overexpression improves water retention, rendering transgenic potato plants more resilient to drought than the WT.

### 2.3. Effects of Drought Stress on Photosynthetic Characteristics in Potato

**Figure 4:**
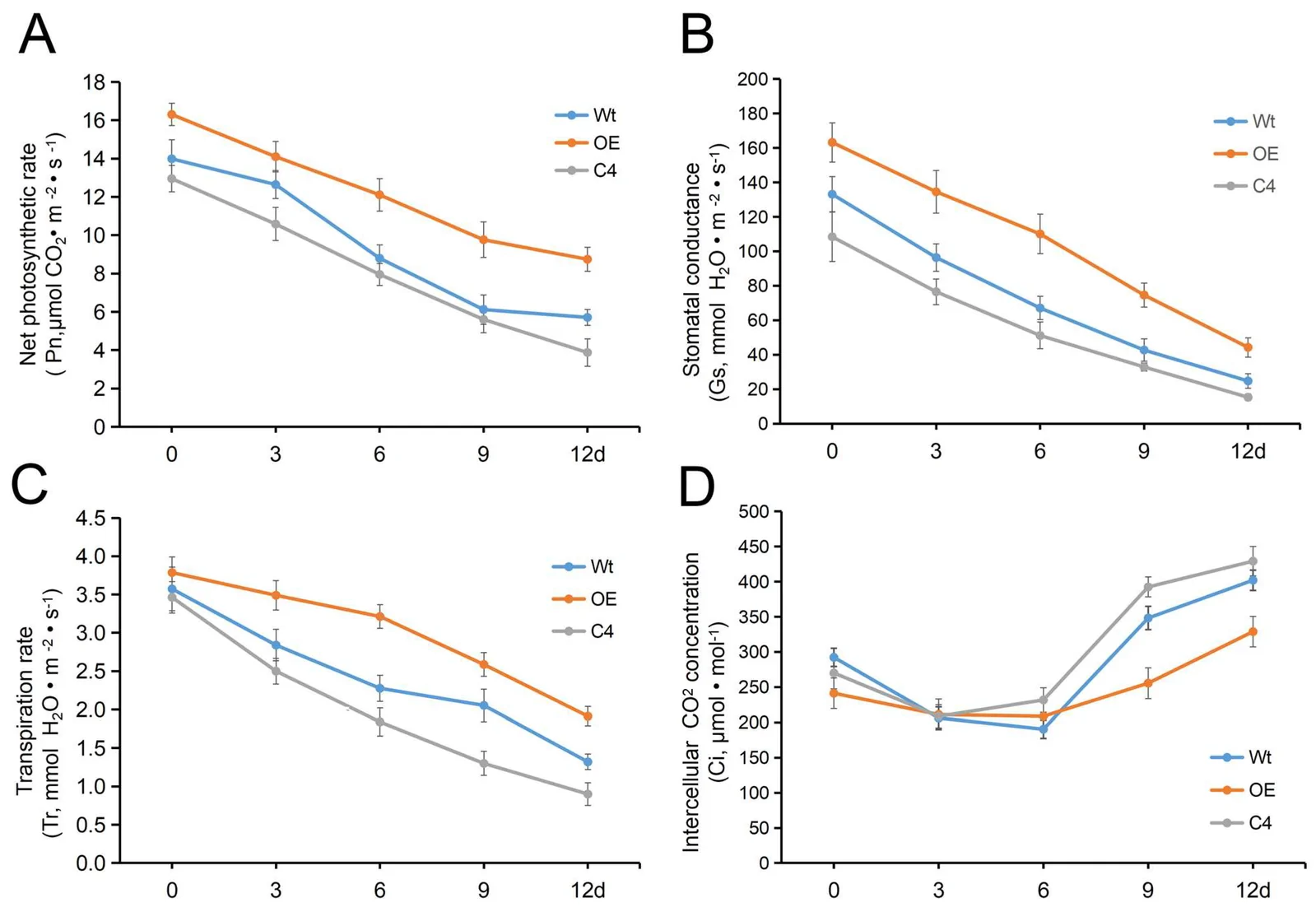
Changes in photosynthetic characteristics of transgenic and WT potatoes under drought stress. (**A**) Net photosynthetic rate; (**B**) Stomatal conductivity; (**C**) Transpiration rate; (**D**) Intercellular CO_2_ concentration.

Drought stress imposed a significant inhibitory effect on the leaf gas exchange capacity across all lines. Over the course of the 12-day drought treatment, all plants exhibited marked reductions in net photosynthetic rate (*Pn*), stomatal conductance (*Gs*), and transpiration rate (*Tr*), which were accompanied by a concomitant rise in intercellular CO_2_ concentration (*Ci*) (Figure 4A–D).

Throughout the drought treatment, the OE plants consistently maintained higher photosynthetic efficiency throughout the stress period. By day 12 of drought, the *Pn* of OE lines remained at 8.74 μmol·m^−2^·s^−1^, which was 1.53-fold of the WT. Furthermore, the OE lines exhibited a significantly more gradual increase in Ci compared to other lines, suggesting a more robust carboxylation efficiency under water deficit conditions. In contrast, the C4 plants were the most severely affected, displaying the lowest photosynthetic capacity. At the end of the treatment, the *Pn*, *Gs*, and *Tr* in C4 plants decreased by 70%, 86%, and 74%, respectively, relative to their initial values. Notably, the C4 lines reached the highest *Ci* levels (429.20 μmol·mol^−1^), indicating that the decline in their photosynthetic rate was likely driven by non-stomatal limitations, such as damage to the photosynthetic apparatus, rather than mere stomatal closure.

### 2.4. Effects of Drought Stress on Antioxidant System and Osmotic Regulatory Substances

**Figure 5:**
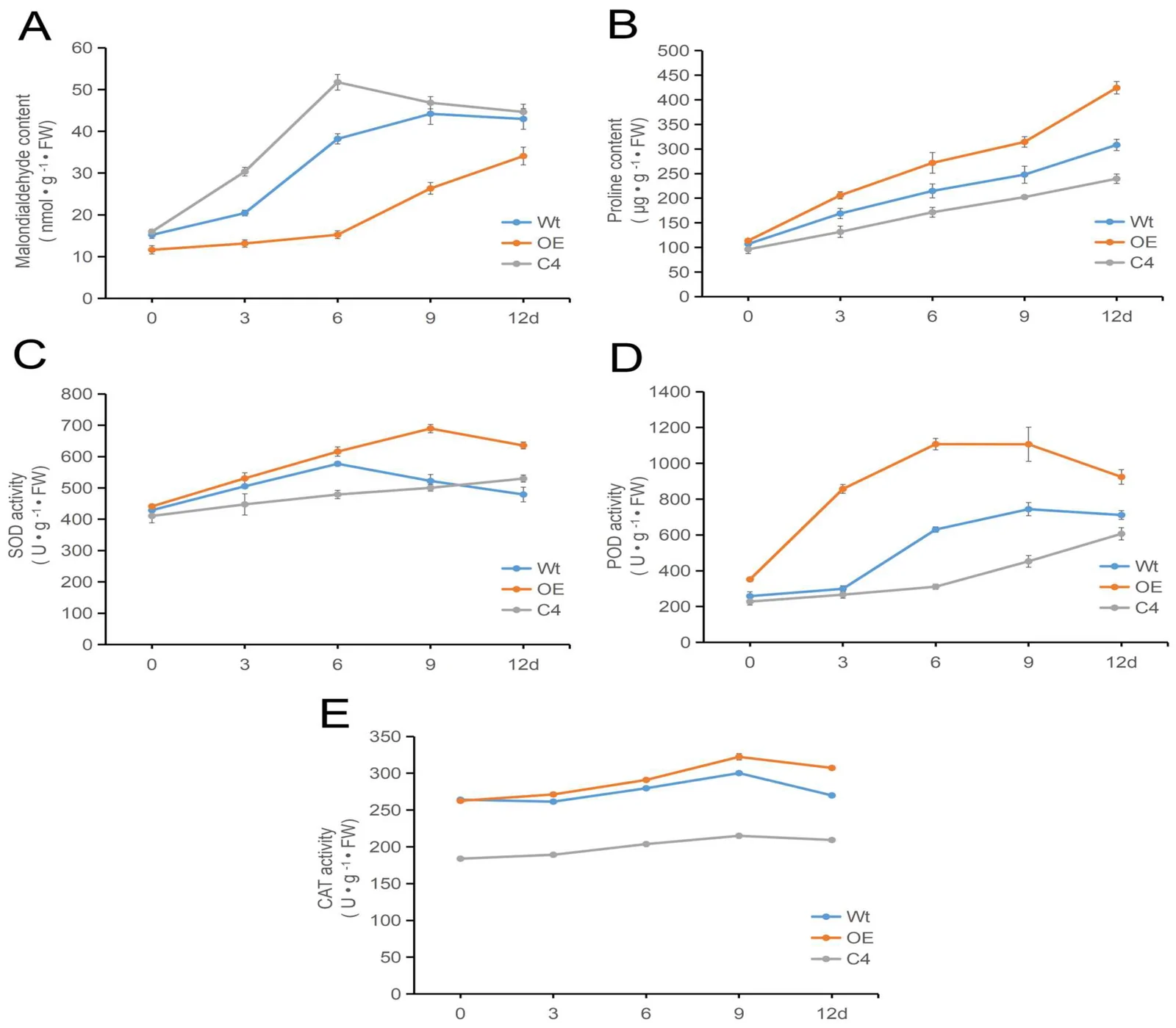
Effects of drought stress on physiological indicators in transgenic and WT potatoes leaves. (**A**) Malondialdehyde content, (**B**) proline content, (**C**–**E**) activities of superoxide dismutase (SOD), peroxidase (POD), and catalase (CAT).

As drought stress continued, malondialdehyde (MDA) content in leaves initially increased and subsequently decreased (Figure 5A). OE plants accumulated the least MDA, reaching 2.26-fold of control levels on day 9, significantly lower than WT (2.90-fold) and C4 (2.93-fold). Proline (*Pro*) level significantly increased under drought conditions (Figure 5B). OE plants showed the most rapid and highest accumulation of *Pro*, reaching 1.77-fold of the control on day 9, significantly higher than WT and C4.

Activities of three antioxidant enzymes, superoxide dismutase (SOD), peroxidase (POD), and catalase (CAT), increased under drought stress (Figure 5C–E). OE plants consistently maintained the highest enzyme activities, with the greatest increases, whereas C4 plants exhibited the lowest enzyme activities. On day 9 of drought treatment, activities of SOD, POD, and CAT in OE plants increased by 1.56-, 3.14-, and 1.22-fold, respectively, compared to control, significantly higher than WT and C4.

### 2.5. Transcriptome Sequencing (RNA-Seq) Data Overview and DEG Analysis

**Figure 6:**
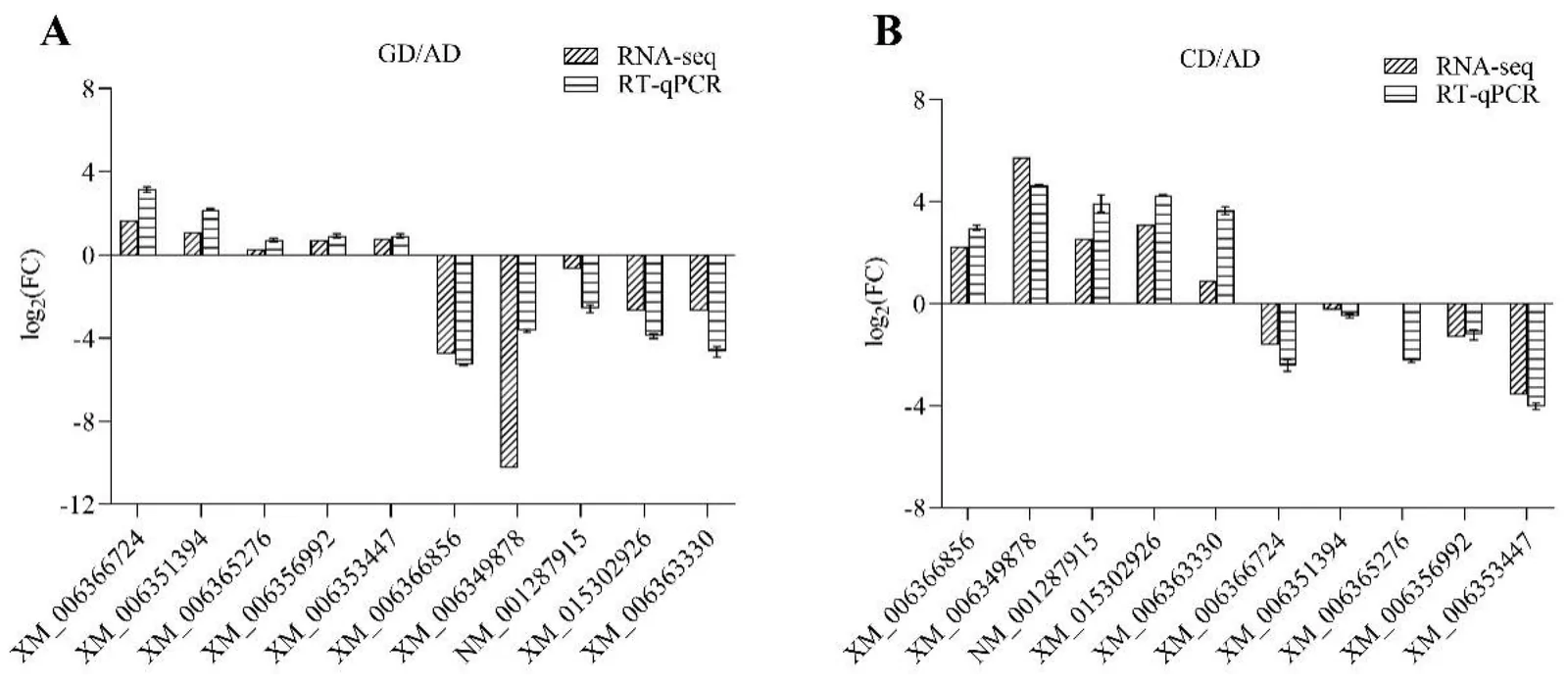
RNA-seq data and RT-qPCR validation of 10 random genes. (**A**) Related expression value of 10 random genes in OE plants comparing with WT under drought stress (GD vs. AD), (**B**) related expression value of 10 random genes expression in C4 plants comparing with WT under drought stress (CD vs. AD). The related expression values were finally calculated to log_2_(FC).

**Figure 7:**
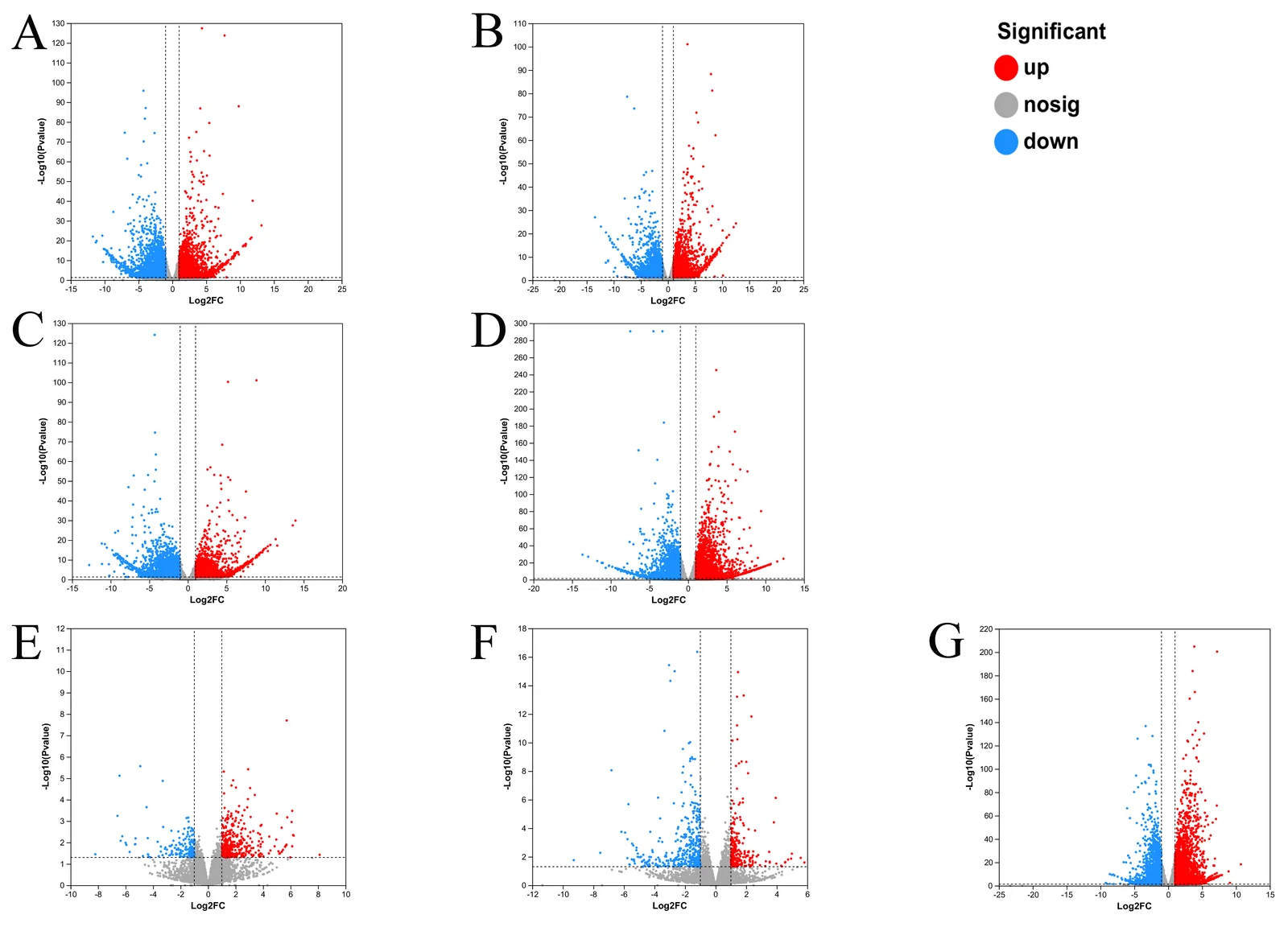
Volcano plots of DEGs. (**A**) G&A; (**B**) C&A; (**C**) GD&AD; (**D**) CD&AD; (**E**) AD&A; (**F**) GD&G; (**G**) CD&C.

RNA-seq was performed on 18 samples on day 9 of drought treatment (see Abbreviations), yielding 854.8 Gb of high-quality clean data. The Q_30_ score for each sample was greater than 95.3%, with alignment efficiencies to the reference genome ranging from 77.91% to 84.22%. RT-qPCR validation of 10 randomly selected differentially expressed genes (DEGs) showed expression trends highly consistent with RNA-seq data (Figure 6), confirming reliability of the transcriptome analysis. Based on transcriptome data, 5520 and 4984 DEGs were identified under normal conditions for OE vs. WT (G&A) and C4 vs. WT (C&A), respectively. Under drought stress, 4793 and 6142 DEGs were identified for OE vs. WT (GD&AD) and C4 vs. WT (CD&AD), respectively. Comparing samples before and after drought stress, 546, 659, and 5685 DEGs were identified for WT (AD&A), OE (GD&G), and C4 (CD&C), respectively (Figure 7).

### 2.6. Transcriptomic Reprogramming of Growth and Defense Pathways

To further elucidate the molecular mechanisms underlying the enhanced growth and photosynthetic capacity of OE plants, Kyoto Encyclopedia of Genes and Genomes (KEGG) pathway enrichment analysis was performed on the DEGs arising specifically from the genetic divergence between OE (G) and WT (A) lines. The analysis revealed overexpression of *StuPPO9* triggered a profound reprogramming of several fundamental metabolic and signaling pathways.

Notably, energy metabolism-related pathways were highly represented, specifically Photosynthesis antenna proteins (37 genes), Photosynthesis (40 genes), and Carbon fixation in photosynthetic organisms (27 genes). The transcriptional activation of these pathways is highly consistent with the superior photosynthetic parameters observed in OE lines.

**Figure 8:**
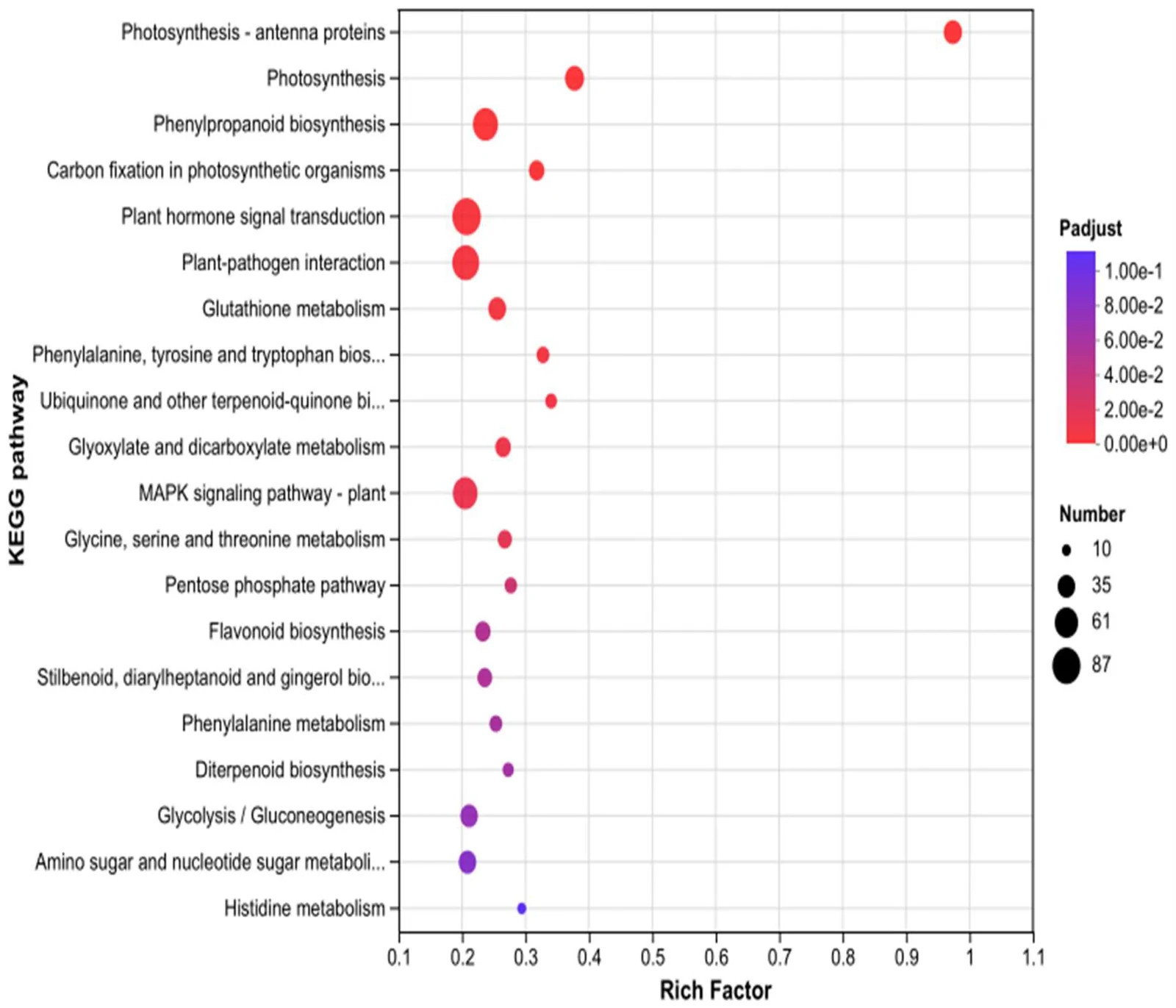
A proposed molecular model of *StuPPO9*-mediated trade-off between growth signaling and secondary metabolism under drought stress.

Furthermore, a substantial suite of DEGs was enriched in plant hormone signal transduction (87 genes, predominantly associated with auxin signaling) and the MAPK signaling pathway (66 genes), which likely serves as the molecular basis for the more robust root systems and enhanced plant vigor. Additionally, key secondary metabolic processes, including Phenylpropanoid biosynthesis (68 genes) and Flavonoid biosynthesis (27 genes), were significantly modulated. These findings suggest that *StuPPO9* plays a pivotal role in orchestrating a trade-off between secondary metabolism and growth-related signaling to promote overall plant fitness (Figure 8).

**Figure 9:**
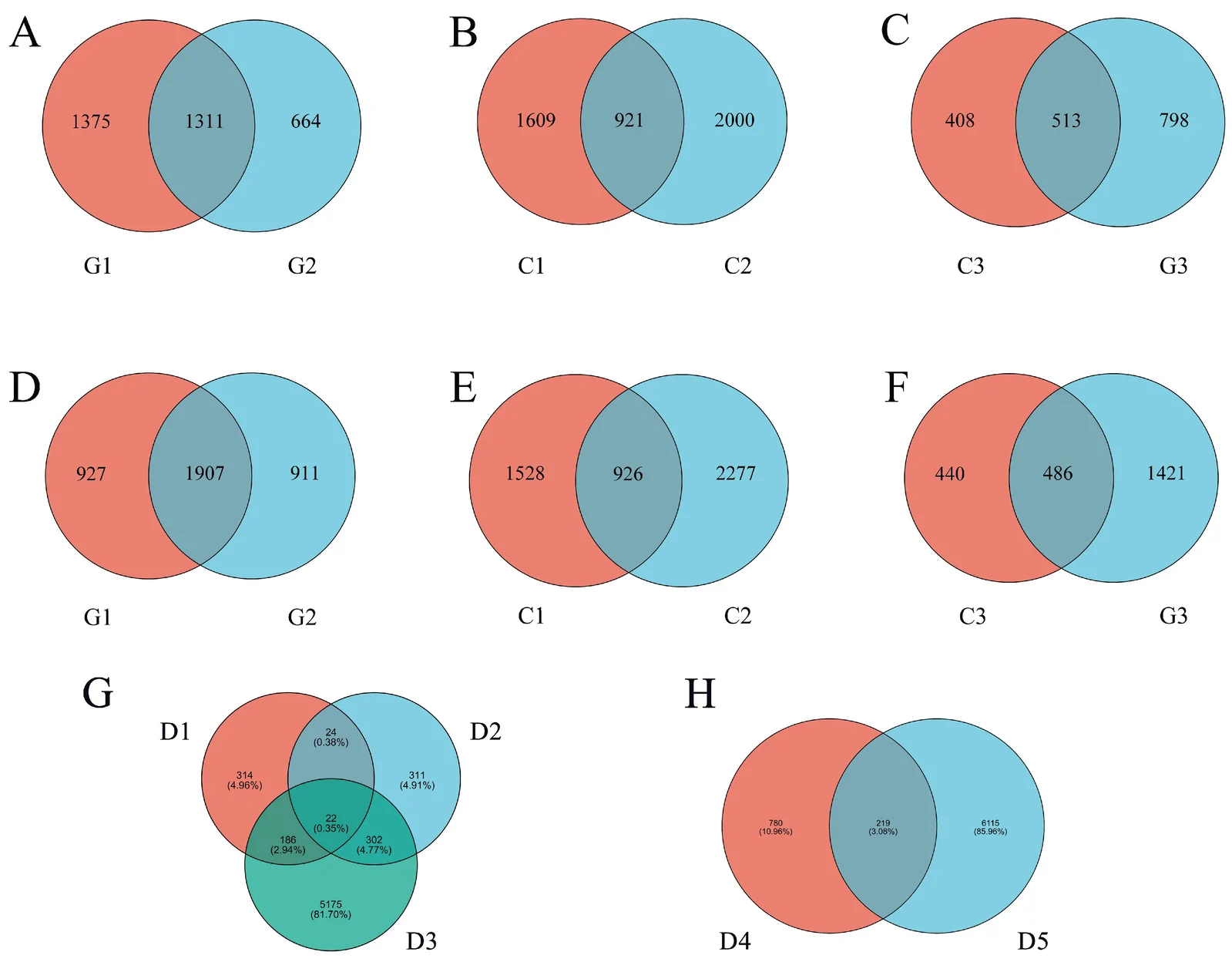
Venn diagrams of DEGs in transgenic potatoes under drought conditions. Note: G1: G&A; G2: GD&AD; G3: G1 ∩ G2; C1: C&A; C2: CD&AD; C3: C1 ∩ C2; D1: AD&A; D2: GD&G; D3: CD&C; D4: C3 ∩ G3; D5: D1∪D2∪D3. (**A**) G1&G2 (1311 upregulated DEGs in OE under drought stress); (**B**) C1&C2 (921 downregulated DEGs in C4 under drought stress; (**C**) C3&G3 (513 DEGs that were upregulated in OE but downregulated in C4 under drought stress); (**D**) G1&G2 (1907 downregulated DEGs in OE under drought stress; (**E**) C1&C2 (926 upregulated DEGs in C4 under drought stress; (**F**) C3&G3 (486 DEGs that were downregulated in OE but upregulated in C4 under drought stress); (**G**) D1&D2&D3; (**H**) D4&D5.

Venn analysis identified 513 DEGs that were upregulated in OE but downregulated in C4 under drought stress (Figure 9C). Conversely, 486 DEGs exhibited the opposite pattern—upregulated in C4 and downregulated in OE (Figure 9F)—resulting in a total of 999 *StuPPO9*-related DEGs with contrasting expression profiles. Meanwhile, a pool of 6334 drought-responsive DEGs was identified by consolidating non-redundant DEGs from OE, C4, and WT plants under stress conditions (Figure 9G). The intersection of these 6334 genes with the 999 *StuPPO9*-related DEGs yielded 219 key DEGs that are both regulated by *StuPPO9* and involved in the drought stress response (Figure 9H).

**Figure 10:**
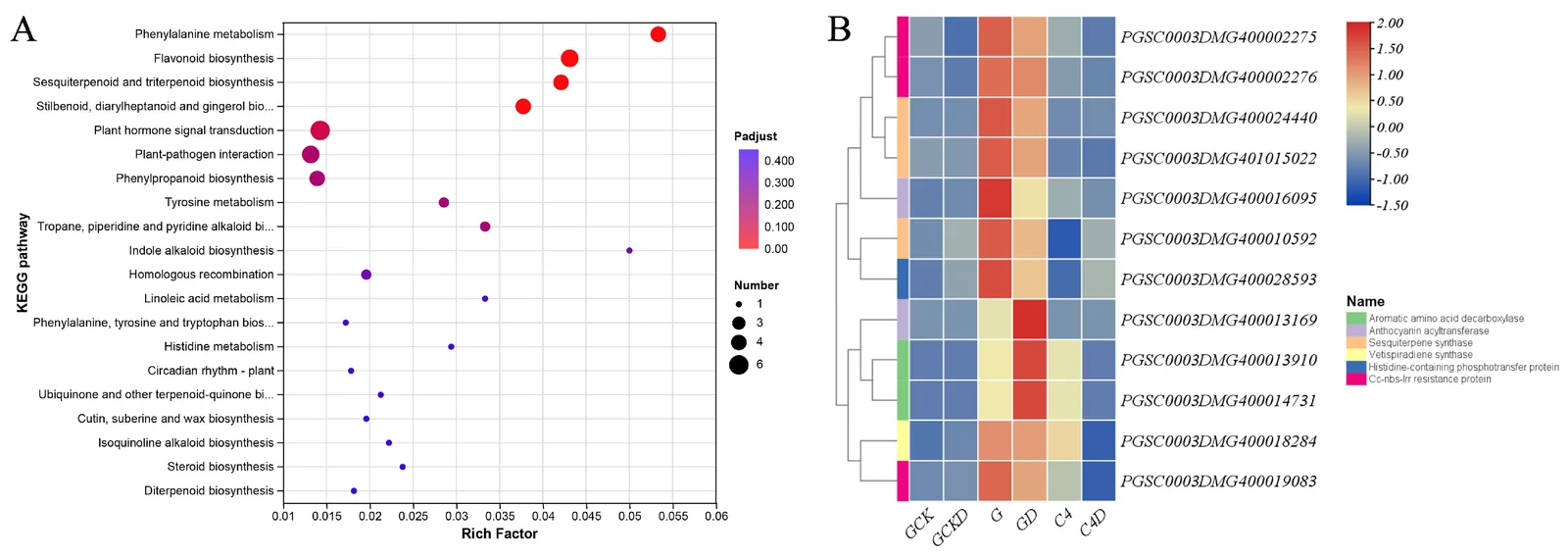
Kyoto Encyclopedia of Genes and Genomes (KEGG) pathway enrichment and expression analysis of key DEGs regulated by *StuPPO9* under drought stress. (**A**) Bubble plot of KEGG pathway enrichment analysis of 219 genes. (**B**) Heatmap of key gene expression levels.

KEGG pathway enrichment analysis of the 219 key DEGs indicated that *StuPPO9* predominantly influenced pathways related to phenylpropanoid metabolism (phenylalanine metabolism, phenylpropanoid biosynthesis, flavonoid biosynthesis), terpenoid metabolism (sesquiterpene and triterpene biosynthesis, diterpene biosynthesis), hormone signaling, and plant–pathogen interactions under drought stress (Figure 10A). Key genes involved in these pathways, as shown in the heatmap, were significantly upregulated in OE plants, including histidine phosphotransfer protein (*HPt*), anthocyanin acyltransferase, sesquiterpene synthase, and vetispiradiene synthase. Under drought stress, these genes maintained high expression levels in OE plants compared with WT and C4 plants. Additionally, aromatic amino acid decarboxylase 2 and anthocyanin acyltransferase exhibited the highest expression levels in OE plants during drought (Figure 10B).

## 3. Discussion

This study systematically demonstrated the crucial role of the potato *StuPPO9* gene in drought stress response. Overexpression of this gene significantly improved plant growth maintenance and stabilized root architecture under drought conditions. Importantly, through integrated transcriptomic, physiological, biochemical, and phenotypic analyses, the underlying synergistic regulatory network was elucidated.

Key upregulated genes identified by transcriptomic analysis, including anthocyanin acyltransferase and aromatic amino acid decarboxylase 2 (phenylpropanoid metabolism), sesquiterpene synthase and vetispiradiene synthase (terpenoid biosynthesis), and *HPt* (signaling pathways), do not act in isolation but form a multi-layered defense network. The upregulation of these genes highlights three core drought adaptation strategies. Firstly, *StuPPO9* triggers a robust metabolic shift within the phenylpropanoid pathway, specifically prioritizing phenylalanine metabolism and flavonoid biosynthesis. This repartitioning of metabolic flux, marked by the upregulation of anthocyanin acyltransferase, favors the accumulation of flavonoids and anthocyanins over lignin biosynthesis [15]. Such a shift significantly augments ROS scavenging capacity and preserves cellular redox homeostasis [16], thereby enabling OE plants to sustain growth under oxidative stress. Secondly, the elevated expression of sesquiterpene and vetispiradiene synthases suggests a targeted activation of the terpenoid biosynthesis pathway. This mechanism mirrors the function of *TgHPTPS2* in *Torreya grandis* and *SsdTPS* in *Arabidopsis*, where terpenoid modulation directly alleviates drought-induced damage by scavenging ROS or indirectly by fine-tuning ABA signaling [17,18]. Furthermore, the potential synergistic interaction between accumulated sesquiterpenes and diterpene oxidation products may provide an additional layer of protection against severe dehydration [19]. Thirdly, drought stress upregulates the HPt protein in hormone signaling pathways. As a component of intracellular nucleotide phosphotransfer signaling [20], increased *HPt* expression suggests that *StuPPO9* modulates phosphorelay signaling cascades, thus regulating downstream stress-responsive genes. In Arabidopsis, *AHP2*, *AHP3*, *AHP4*, and *AHP5* have established roles in drought response, regulating processes such as stomatal function and ROS scavenging [21]. Additionally, high expression of aromatic amino acid decarboxylase 2 may contribute to the synthesis of phenylpropanoid derivatives such as 4-ethylphenol, involved in cell wall modification and stress signaling [22]. In the plant–pathogen interaction pathway, accumulated phenylpropanoid and terpenoid metabolites may enhance cell wall integrity and antimicrobial compound synthesis [23]. Collectively, these findings demonstrate that *StuPPO9* enhances drought tolerance through a multi-layered molecular network involving coordinated carbon allocation, reprogramming of phenylpropanoid and terpenoid metabolic fluxes, integration of hormone signaling, and reinforcement of cell wall structures.

This series of molecular reprogramming directly translates into physiological advantages. For example, the activation of phenylpropanoid and terpenoid metabolism, along with enhanced antioxidant enzyme (SOD, POD, CAT) activities [24,25], constitutes a robust defense mechanism against oxidative damage in OE plants, as evidenced by their lowest MDA accumulation. Meanwhile, metabolic flux adjustments may be associated with nitrogen metabolism, promoting rapid accumulation of proline, thus achieving effective osmotic regulation [26]. These physiological improvements create favorable conditions for maintaining photosynthetic stability. Specifically, the strengthened antioxidant system protects photosystem II, while more precise stomatal regulation, potentially mediated by hormonal signaling (reflected in slower declines in *Gs* and slower increases in *Ci*) [20,27], enables OE plants to better balance water conservation and carbon fixation, thus maintaining higher *Pn*.

This optimized system, from molecular to physiological levels, clearly manifests in the overall drought resistance phenotype. A well-developed root system and strengthened cell walls enhance water acquisition and retention [28]. Efficient photosynthesis and metabolic homeostasis provide carbon skeletons and energy for sustained growth [29,30,31]. Robust oxidative stress tolerance ensures cell vitality and functional integrity under drought conditions [32]. Therefore, the integrated drought-resistant phenotypes exhibited by OE plants, such as reduced growth inhibition, delayed wilting, and smaller leaf area reductions, are outcomes of adaptive responses triggered by the internal gene expression network reshaped by *StuPPO9*.

It should be noted that this study evaluated a single representative OE line and CRISPR knockdown line due to poor vigor of other independent line candidates during the transplantation and acclimation phases. While the highly consistent multi-layered evidence—spanning transcriptomic profiles, secondary metabolic fluxes, physiological markers, and whole-plant phenotypes—strongly supports the key regulatory role of *StuPPO9*, the possibility of line-specific position effects or off-target mutations cannot be completely ruled out. Future studies incorporating additional independent transgenic lines will be important to further solidify the functional characterization of *StuPPO9* under drought stress.

## 4. Materials and Methods

### 4.1. Vector Construction and Identification of Transgenic Potatoes

To generate the overexpression (OE) construct, the full-length coding sequence (CDS) of *StuPPO9* (GenBank ID: XM_006347021.2) was amplified and cloned into the pCAMBIA1301S vector at the *BamH* I and *Pst* I restriction sites [9]. The recombinant plasmids were transformed into *Escherichia coli* strain *DH5α*, and the accuracy of the construct, designated as pCAMBIA1301S-*StuPPO9*, was verified by Sanger sequencing. For CRISPR/Cas9-mediated knockout, two single-guide RNAs targeting specific exons (Target sites 1 and 2, Figure 1) were designed and integrated into the pHSN401 vector.

All constructs were introduced into *Agrobacterium tumefaciens* strain GV3101 and subsequently transformed into the potato cultivar ‘Atlantic’ via the hypocotyl transformation method. Putative transgenic lines were screened on MS medium supplemented with 50 mg/L kanamycin for OE lines and 10 mg/L hygromycin for CRISPR/Cas9 lines. Positive OE lines were identified by PCR amplification of the *GUS* gene, while knockout (C4) lines were confirmed by detecting the *HptII* gene. The mutation patterns at the target sites in C4 lines were further characterized by sequencing and sequence alignment analysis using primers of sgRNA S and sgRNA X. *StuPPO9*-specific primers (*StuPPO9*-F/R) were designed for RT-qPCR to confirm *StuPPO9* expression levels. All primer sequences used in this study are listed in Supplementary Table S1.

### 4.2. Drought Treatment

Potato plantlets of WT, OE, and C4 lines were grown in pots (9 cm diameter) filled with a mixture of commercial peat moss substrate (Pindstrup Substrate, Denmark; pH 5.5–6.0, organic matter content ≥ 85%) and vermiculite (3:1, *v*/*v*). After 20 days of growth, seedlings of similar size and exhibiting vigorous growth were selected for the experiment. Before treatment, all plants were well-watered to maintain the soil relative water content (SRWC) at 75–80% of field capacity. For the constant drought treatment, watering was restricted until the SRWC reached 50%, and this level was strictly maintained for 12 days by weighing the pots daily at 18:00 and replenishing the lost water with the calculated amount of distilled water. A separate control group (CK) was maintained at 75–80% SRWC throughout the 12 days for morphological comparison (presented in Table 1). For physiological and photosynthetic analyses during the drought progression, measurements were taken at 3-day intervals (0, 3, 6, 9, and 12 days after the initiation of drought), with day 0 (pre-treatment, 75–80% SRWC) serving as the baseline control. Each treatment was conducted in triplicate, with 10 pots per replicate, totaling 30 plants per genotype.

### 4.3. Measurement of Morphological Growth

At the end of the 12-day drought treatment, the morphological growth parameters of the WT, OE, and C4 lines were recorded. Plant length was measured from the soil surface to the apical meristem using a graduated ruler. Stem diameter was determined at the base of the stem (approximately 1 cm above the soil) using a digital vernier caliper. The single leaf area was estimated using the manual measurement method. Specifically, the maximum length (L) and maximum width (W) of each leaf were measured using a graduated ruler, and the leaf area was calculated using the formula: Area = L × W × k, where k is the shape correction coefficient (set at 0.75 for potato leaves in this study). The net changes in growth parameters, including plant length, stem diameter, and single leaf area, were determined using the following equation:

△*P* = *P*_12_ − *P*_0_

where △*P* represents the net change in the respective growth parameter (length, diameter, or leaf area); *P*_12_ denotes the value recorded at the conclusion of the 12-day drought treatment. *P*_0_ denotes the initial value measured at day 0 (baseline), representing the onset of the experiment.

Relative water content (RWC) of potato leaves and roots was determined according to the standard physiological methods [33] with minor modifications. For leaf RWC, fully expanded the fourth leaf leaves from the top were strictly collected from identical chronological and developmental positions across both control and drought treatments to eliminate confounding effects of leaf age. For root RWC, the entire root system was carefully harvested, washed gently with tap water to remove residual soil or substrate, and thoroughly blotted dry with paper towels. The measurement process for both tissues was conducted as follows: Immediately after harvesting, the samples were weighed to obtain the fresh weight (FW). The samples were then fully immersed in deionized water in closed Petri dishes and keep at 4 °C in the dark for 24 h. After incubation, excess surface water was gently blotted off using filter paper, and the samples were weighed immediately to obtain the turgid weight (TW). Finally, the samples were placed in paper bags, oven-dried at 105 °C for 30 min for enzyme inactivation, and then dried a 80 °C to a constant weight (approximately 48 h) to determine the dry weight (DW). The relative water content (RWC) was calculated using the following equation:

$$\mathrm{RWC}(\%)=\frac{\mathrm{FW}-\mathrm{DW}\;}{TW-DW}\times 100$$

where FW is the fresh weight of the leaf or root sample (g); TW is the turgid weight after rehydration (g); DW is the constant dry weight after oven-drying (g).

For plant length, stem diameter, leaf area and RWC measurement, to ensure that leaves from different treatments (control vs. drought) were at fully comparable developmental and physiological stages, measurements were strictly performed on leaves sampled from identical node positions, specifically the fourth fully expanded functional leaves from the top of each plant. For each biological replicate, leaves were sampled from three independent plants and averages (totaling 9 individual leaves across three biological replicates per genotype) to guarantee representative statistical data.

To assess root system morphological changes under control (CK) and drought stress conditions, the roots of potato plants were carefully excavated and analyzed. Potting substrate was gently washed away from the root system using a low-pressure water stream over a fine sieve to avoid any loss of fine lateral roots. The primary roots (directly originating from the stem base) were manually counted. The first-order lateral roots branching directly from the primary roots were systematically counted for each plant. For each genotype, three independent individual plants were evaluated as three biological replicates (*n* = 3). For each biological replicate, the entire intact root system was comprehensively and systematically counted to determine the average root numbers.

### 4.4. Measurement of Photosynthetic Parameters

Net photosynthetic rate (*Pn*), stomatal conductance (*Gs*), transpiration rate (*Tr*) and intercellular CO_2_ concentration (*Ci*) were measured using a portable photosynthesis system (CI-340, CID Bio-Science, Camas, WA, USA). The measurements were performed between 10:00 AM and 11:00 AM on the fourth fully expanded functional leaf from the top of each plant to avoid the effects of midday depression. To ensure that leaves from both control and drought treatments were at identical and fully comparable physiological and developmental stages, the target leaves were strictly matched by node position and leaf age across all plant lines. During the measurements, the light intensity was set to a constant photosynthetically active radiation (PAR) of 1000 μmol·m^−2^·s^−1^, the leaf temperature was maintained at 25 ± 2 °C, and the ambient CO_2_ concentration was approximately 400 ± 10 μmol·mol^−1^. For each treatment, three plants were randomly selected as biological replicates. For each biological replicate, leaves were sampled from three independent plants and averages (totaling 9 individual leaves across three biological replicates per genotype) to guarantee representative statistical data.

### 4.5. Measurements of Physiological Indices

To minimize variations caused by tissue maturity and spatial differences, leaf samples used for physiological assays were harvested from the exact same node positions (specifically the third and fourth fully expanded functional leaves). For all physiological indices, three samples were randomly selected as biological replicates for each treatment.

Malondialdehyde (MDA) content was measured using the thiobarbituric acid (TBA) reactive substances (TBARS) method. Briefly, fresh leaf samples (0.5 g) were homogenized in 5 mL of 5% (*w*/*v*) trichloroacetic acid (TCA) and centrifuged at 10,000× *g* for 15 min at 4 °C. The supernatant (2 mL) was mixed with 2 mL of 0.6% (*w*/*v*) TBA (prepared in 20% TCA). The mixture was heated in a boiling water bath (100 °C) for 30 min, quickly cooled on ice, and then centrifuged again. Absorbance was recorded at 450, 532, and 600 nm. The MDA concentration (C, μmol/L) was calculated using the following formula:

C = 6.45 × (A_532_ − A_600_) − 0.56 × A_450_

The final MDA content was expressed as μmol/g FW (Fresh Weight).

Free proline content was measured using the acid ninhydrin method. Approximately 0.3 g of leaf tissue was extracted in 5 mL of 3% (*w*/*v*) sulfosalicylic acid in a boiling water bath for 10 min. After cooling and filtering, 2 mL of the filtrate was reacted with 2 mL of acid ninhydrin and 2 mL of glacial acetic acid at 100 °C for 1 h. The reaction was terminated on ice, and the chromophore was extracted with 4 mL of toluene. The absorbance of the organic phase was measured at 520 nm using toluene as a blank. The concentration of proline (C_pro_) was determined from a standard curve (y = ax + b) prepared with L-proline. The content was calculated as follows:

$$\mathrm{Proline}\;\mathrm{content}\;(\mu g/\mathrm{gFW})\;=\;\frac{C_{\mathrm{pro}}\;\times \;V}{W}$$

where V is the total volume of the sulfosalicylic acid extract (mL) and W is the sample weight (g).

Antioxidant enzyme activities, including superoxide dismutase (SOD), peroxidase (POD), and catalase (CAT), were analyzed by homogenizing 0.5 g of fresh tissue in 5 mL of 50 mM ice-cold phosphate buffer (pH 7.8) containing 1% (*w*/*v*) polyvinylpolypyrrolidone (PVPP). The homogenate was centrifuged at 12,000× *g* for 20 min at 4 °C, and the resulting supernatant was used for enzyme assays [34]. Superoxide dismutase (SOD) activity was determined by its ability to inhibit the photochemical reduction of nitroblue tetrazolium (NBT) at 560 nm, where one unit of SOD is defined as the amount of enzyme causing 50% inhibition. Peroxidase (POD) activity was assayed using the guaiacol method, monitoring the increase in absorbance at 470 nm due to guaiacol oxidation (ϵ = 26.6 mM^−1^ cm^−1^). Catalase (CAT) activity was measured by following the consumption of H_2_O_2_ at 240 nm (ϵ = 39.4 M^−1^ cm^−1^). All assays were conducted with three biological replicates.

$$\mathrm{SOD}\;\mathrm{Activity}\;(U/\mathrm{gFW})=\frac{(A_{\mathrm{ck}}-A_{E})\times V_{t}}{A_{\mathrm{ck}}\;\times \;0.5\;\times \;W\;\times \;V_{s}}$$

where Ack is the absorbance of the control, AE is the absorbance of the sample, and Vt and Vs are total and assay volumes, respectively.

$$\mathrm{POD}\;\mathrm{or}\;\mathrm{CAT}\;\mathrm{Activity}\;(U/\mathrm{gFW}\cdot \min)=\frac{\Delta A\;\times \;V_{t}}{d\;\times \;\epsilon \;\times \;W\;\times \;V_{s}\;\times \;t}$$

For POD, ΔA is the change at 470 nm, ϵ = 26.6 mM^−1^cm^−1^. For CAT, ΔA is the change at 240 nm, ϵ = 39.4 M^−1^cm^−1^. d represents the cuvette light path (1 cm) and t is the reaction time (min).

### 4.6. Transcriptome Sequencing and Analysis

The fourth fully expanded mature leaf from each potato plant on day 9 of drought treatment was selected. Each biological replicate consisted of a mixture of leaves from five individual plants. Each treatment included three biological replicates, resulting in a total of 18 samples. The six sample groups were listed in the Abbreviations.

Total RNA was extracted from the leaves of WT, OE, and C4 plants under both control and drought stress conditions using the plant RNA extraction kit (TIANGEN, Beijing, China). Transcriptome sequencing on the Illumina HiSeq 2000 platform (Illumina, Inc., San Diego, CA, USA) and data analysis were performed by Shanghai Majorbio Bio-Pharm Techenology Co., Ltd. (Shanghai, China). Library construction, sequencing, and quality control were completed by Majorbio. Raw data were further processed using the Majorbio platform (https://www.majorbio.com, accessed on 15 May 2024). Clean reads were aligned to the potato reference genome (http://solanaceae.plantbiology.msu.edu/pgsc_download.shtml, accessed on 15 May 2024) using HISAT software (v2.1.0) to evaluate alignment efficiency. Gene expression levels were quantified as Fragments Per Kilobase of transcript per Million mapped reads (FPKM). DEG analysis was conducted using the DESeq2v1.30.1 software [35] with selection criteria set at |log_2_FC| ≥ 1, *p*-value < 0.05, and *p*-adjust < 0.01. The False Discovery Rate (FDR) was calculated by adjusting the *p*-value using the Benjamini–Hochberg (BH) method (*p*-adjust). The identified DEGs were subjected to KEGG enrichment analysis to investigate response genes of transgenic and WT potato plants under drought stress.

### 4.7. RT-qPCR Validation

To validate the reliability of the transcriptome data, ten key differentially expressed genes (DEGs) were randomly selected for qRT-PCR analysis. Total RNA was isolated from potato tissues using a plant RNA extraction kit (TIANGEN, Beijing, China), following the manufacturer’s protocols. Subsequently, 1 μg of the purified RNA was reverse-transcribed into cDNA using the PrimeScript™ RT reagent Kit (Takara, Dalian, China). The first step involved genomic DNA removal at 42 °C for 2 min, followed by the synthesis of a 20 μL cDNA library under the conditions of 37 °C for 15 min and 85 °C for 5 s.

The quantitative PCR assays were conducted on a qTower 2.2 system (Analytik Jena GmbH, Jena, Germany) using 96-well plates. Each 25 μL reaction mixture comprised 12.5 μL Premix Ex Taq™ II, 1 μL of each primer (forward and reverse), 2 μL of diluted cDNA template, and 8.5 μL of ddH2O. To ensure accuracy, no-template controls (NTC) were included to monitor primer dimerization, and all samples were analyzed in triplicate. The thermal cycling program consisted of an initial denaturation at 95 °C for 30 s, followed by 40 cycles of 95 °C for 5 s and 60 °C for 30 s. Fluorescence data were captured during the 60 °C annealing/extension phase. *β-tubulin* served as the internal reference. Each sample had three biological replicates, and each biological replicate was measured in three technical replicates. The relative mRNA abundance was quantified using the 2^−ΔΔCt^ method. All specific primer sequences are detailed in Table S1.

### 4.8. Data Analysis

Sample data were analyzed using one-way analysis of variance (ANOVA) with IBM SPSS 26.0 software. Multiple comparisons were performed using the Duncan test with a *p*-value of 0.05 and 0.01, respectively. Graphs were generated using GraphPad Prism 9 software.

## 5. Conclusions

This study demonstrates that *StuPPO9* does not function merely as an isolated “antioxidant enzyme gene,” but rather as a critical regulatory node. By coordinating primary signal transduction, hormone regulation, and secondary metabolism reprogramming, *StuPPO9* systematically enhances potato drought tolerance. This provides a promising target with network-regulatory potential for crop stress resistance breeding. Future research should investigate whether the StuPPO9 protein has non-enzymatic signaling functions and use metabolomics to precisely characterize the metabolite dynamics regulated by *StuPPO9*, providing a more comprehensive understanding of this drought response network.

## Figures and Tables

**Table 1:** Changes in growth indicators of *StuPPO9* transgenic potato under drought stress.

Net Growth Changes	Treatment	WT	OE	C4
Plant length (cm)	CK	20.37 ± 0.79 a	21.13 ± 0.72 a	20.33 ± 0.38 a
	Drought	12.07 ± 0.52 c	14.73 ± 0.46 b	10.30 ± 0.15 d
Stem diameter (mm)	CK	1.51 ± 0.01 b	1.59 ± 0.01 a	1.49 ± 0.01 b
	Drought	1.20 ± 0.03 d	1.32 ± 0.01 c	0.87 ± 0.03 e
Single leaf area (cm^2^)	CK	5.81 ± 0.11 ab	6.02 ± 0.15 a	5.50 ± 0.09 b
	Drought	2.72 ± 0.05 d	4.37 ± 0.18 c	2.16 ± 0.09 e
Leaf relative water content (%)	CK	92.35 ± 0.14 ab	92.94 ± 0.11 a	92.10% ± 0.09 bc
	Drought	90.45 ± 0.26 d	91.55 ± 0.22 c	87.44% ± 0.21 e
Root relative water content (%)	CK	94.30 ± 0.10 a	94.33 ± 0.05 a	93.93% ± 0.10 a
	Drought	91.24 ± 0.24 c	92.76 ± 0.11 b	89.10% ± 0.21 d
Number of primary roots	CK	3.33 ± 0.33 ab	3.67 ± 0.33 a	3.00 ± 0.00 b
	Drought	2.67 ± 0.33 b	3.33 ± 0.33 a	2.33 ± 0.33 b
Number of lateral roots	CK	21.33 ± 1.45 a	14.67 ± 0.88 c	16.67 ± 1.20 b
	Drought	10.33 ± 0.88 d	10.67 ± 1.20 d	8.67 ± 0.67 e

Note: Data are expressed as mean ± SE. The significance of differences in data between the same trait under drought stress treatment is marked with different lowercase letters (*p* < 0.05).

## Data Availability

The raw sequencing data generated in this study have been deposited in the NCBI Sequence Read Archive (SRA) database under accession number [PRJNA1404111]. Other relevant biological data are included within the article and its Supplementary Materials. Further inquiries can be directed to the corresponding author.

## Supplementary Materials

The following supporting information can be downloaded at: https://www.mdpi.com/article/10.3390/plants15162495/s1, Table S1: Information on primer sequences, Figure S1: Sequence alignment and Sanger sequencing chromatograms of *StuPPO9* and five CRISPR/Cas9-induced mutant alleles.

## Abbreviations

WT	wild-type
OE	*StuPPO9*-overexpressing line
C4	CRISPR/Cas9-mediated knockout line
A	WT leaves under control
G	OE leaves under control
C	C4 leaves under control
AD	WT leaves under drought stress
GD	OE leaves under drought stress
CD	C4 leaves under drought stress
RWC	relative water content
*Pn*	net photosynthetic rate
*Gs*	stomatal conductance
*Tr*	transpiration rate
*Ci*	intercellular CO_2_ concentration
MDA	malondialdehyde
*Pro*	proline
SOD	superoxide dismutase
POD	peroxidase
CAT	catalase
