# Supplementary material for: StuPPO9 Enhances Drought Tolerance in Potato (Solanum tuberosum) by Coordinating Photosynthetic Stability and Secondary Metabolism Reprogramming
Source: Plants (Basel). 2026 Aug 18;15(16):2495. doi: 10.3390/plants15162495 (PMC13516414; doi:10.3390/plants15162495)
Supplement: Supplementary file 1 [file plants-15-02495-s001.zip › Figure S1.pdf]

A

```
StuPPO9     (1825) CCCAAGAATGGTTCTGGTGCTGTTAAAATTGGTGG
StuPPO9-1   (204)  CCCAAGAATGGTTCTGGTGCTGTTAAAATTAATGG
StuPPO9-2   (204)  CCCAAGAATGGTTCT--------TAAAATTGGTGG
StuPPO9-3   (204)  CCCAAGAATGGTTCTA-TGCTGTTAAAATTAGTGG
StuPPO9-4   (204)  CCCAAGAATGGTTCTGGTGCTG---AAATTGGTGG
StuPPO9-5   (204)  CCCAAGAATGGTTCTGGTGCTGTTAAAATT--TGG
Consensus   (1825) CCCAAGAATGGTTCTGGTGCTGTTAAAATTGGTGG
```

B

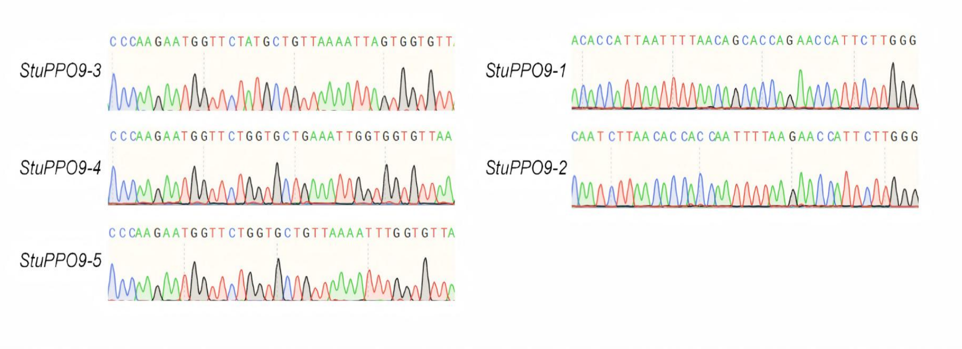

Figure S1. Sequence alignment and Sanger sequencing chromatograms of *StuPPO9* and five CRISPR/Cas9-induced mutant alleles.

A. The top sequence (*StuPPO9*) corresponds to the wild-type reference sequence of potato. *StuPPO9-1* through *StuPPO9-5* indicate five distinct mutant alleles identified in the edited plant C4. The protospacer adjacent motif (PAM, corresponding to 5'-NGG-3' on the reverse strand) is highlighted by a red box (CCC). The 20-bp sgRNA target site is indicated by a line. Hyphens (-) denote nucleotide deletions, and substitutions are indicated by modified nucleotides. Numbers in parentheses indicate the sequence positions. B. Sanger sequencing chromatograms of the target genomic region in the five mutant lines.*StuPPO9-1* and *StuPPO9-2* represent reverse complementary sequences of each other.
