# Supplementary material for: StuPPO9 Enhances Drought Tolerance in Potato (Solanum tuberosum) by Coordinating Photosynthetic Stability and Secondary Metabolism Reprogramming
Source: Plants (Basel). 2026 Aug 18;15(16):2495. doi: 10.3390/plants15162495 (PMC13516414; doi:10.3390/plants15162495)
Supplement: Supplementary file 1 [file plants-15-02495-s001.zip › Table S1.pdf]

Table S1. Information on primer sequences

Gene name	GenBank ID	Forward primer (5'-3')	Reverse primer (3'-5')
GUS	XM_016657042.1	CTTATGTGTAGTCGCTGGTTGA	AAGAGTTGCGCTCAAACGAT
Hyg	NM_001324874.1	AAGGCTCTCCTCTCTGATCC	CTCTTCCTCCTATCGCAAGC
StuPPO9	XM_006347021.2	GGACCCGACGTTACCAAATG	TGATGGAAGCTGGAAGTCGA
sgRNA(S)	XM_006347021.2	ATGTTCATGAATACATCTCAAACAAG	TGTCGATGGGTGAGTTATTGTGT
sgRNA(X)	XM_006347021.2	TTCGAAACGAAAAACGAAGCTGA	AGAAAACACCGTATCTCCACGAA
StMYB48	XM_006366856	GCAAGGGAAGTATCCGGGAG	GGCGTGAAATCCACATCAGG
StTD	XM_006366724	CCAAAACATGCGTGGCTCAA	CCCCCGTTGGGTTATTCACT
StGDSL	XM_006351394	GTTGCCGTTTTTACCGCCTT	TGCGTTTGAAGCGATTGTGG
StCHS	XM_006349878	TGGTTGCGCAAGCTCTCTT	AACCAATAATTGCGTGTTCGCTA
St gdhA	XM_006365276	ACCAGTTGATCTTGGCGGTT	CCTACATTCCCGAACCCCTG
StCYP450	NM_001287915	ACCAAATTTAGATGGGAAGTCGAAG	GGTGGCATCTTGTTTTCTAGGG
StSOX	XM_006356992	CCCCGAAGACTACTACCCGA	ACACGAACAGGGATCGAGTTT
StMIOX	XM_006353447	GGACAAAGAGAACATGAAGTGGC	ACTCTGCCACCATGGATTCAA
StCML19	XM_015302926	GGCGAGTCAACCTCCATTGAT	AACAAGCCCAAGGCCAAAGA
StKCS3	XM_006363330	ATGCTTAGCTTTGGAGCTGGA	TGTGTCATCTTCATCATGTAGCCAT
β-tubulin	Z33382	ATGTTCAGGCGCAAGGCTT/	TCTGCAACCGGGTCATTCAT
